# Runs of homozygosity in a selected cattle population with extremely inbred bulls: Descriptive and functional analyses revealed highly variable patterns

**DOI:** 10.1371/journal.pone.0200069

**Published:** 2018-07-09

**Authors:** Daniel Goszczynski, Antonio Molina, Ester Terán, Hernán Morales-Durand, Pablo Ross, Hao Cheng, Guillermo Giovambattista, Sebastián Demyda-Peyrás

**Affiliations:** 1 IGEVET–Instituto de Genética Veterinaria "Ing. Fernando N. Dulout” (UNLP-CONICET LA PLATA), Facultad de Ciencias Veterinarias UNLP, La Plata, Argentina; 2 Departamento de Genética, Universidad de Córdoba, Córdoba, España; 3 Department of Animal Science, University of California, Davis, Davis, California, United States of America; 4 Departamento de Producción Animal, Facultad de Ciencias Veterinarias, Universidad Nacional de La Plata, La Plata, Buenos Aires, Argentina; Gaziosmanpasa University, TURKEY

## Abstract

The analysis of runs of homozygosity (ROH), using high throughput genomic data, has become a valuable and frequently used methodology to characterize the genomic and inbreeding variation of livestock and wildlife animal populations. However, this methodology has been scarcely used in highly inbred domestic animals. Here, we analyzed and characterized the occurrence of ROH fragments in highly inbred (HI; average pedigree-based inbreeding coefficient F_PED_ = 0.164; 0.103 to 0.306) and outbred Retinta bulls (LI; average F_PED_ = 0.008; 0 to 0.025). We studied the length of the fragments, their abundance, and genome distribution using high-density microarray data. The number of ROH was significantly higher in the HI group, especially for long fragments (>8Mb). In the LI group, the number of ROH continuously decreased with fragment length. Genome-wide distribution of ROH was highly variable between samples. Some chromosomes presented a larger number of fragments (BTA1, BTA19, BTA29), others had longer fragments (BTA4, BTA12, BTA17), while other ones showed an increased ROH accumulation over specific loci (BTA2, BTA7, BTA23, BTA29). Similar differences were observed in the analysis of 12 individuals produced by a similar inbred event (F_PED3_ = 0.125). The correlation between the fraction of the genome covered by ROH (F_ROH_) and F_PED_ was high (0.79), suggesting that ROH-based estimations are indicative of inbreeding levels. On the other hand, the correlation between F_PED_ and the microsatellite-based inbreeding coefficient (F_MIC_) was only moderate (r = 0.44), suggesting that STR-based inbreeding estimations should be avoided. Similarly, we found a very low correlation (r = -0.0132) between recombination rate and ROH abundance across the genome. Finally, we performed functional annotation analyses of genome regions with significantly enriched ROH abundance. Results revealed gene clusters related to pregnancy-associated proteins and immune reaction. The same analysis performed for regions enriched with recently formed ROH (> 8 Mb) showed gene clusters related to flagellum assembly. In both cases, the processes were related to male and female reproductive functions, which may partially explain the reduced fertility associated with inbred populations.

## Introduction

Inbreeding depression is the reduced survival and fertility of offspring from related individuals. According to Leroy [1], the decreased fitness could be mediated by three different genetic mechanisms: 1) an increased phenotypic expression of recessive deleterious mutations (dominance hypothesis); 2) the lack of phenotypic advantages provided by heterozygote genotypes at loci that are maintained by balancing selection at intermediate frequencies (overdominance hypothesis); and 3) an epistatic interaction in which the probability of favourable gene combinations for heterozygotes is higher. However, Charlesworth and Willis [2] proposed the main cause of inbreeding depression to be the dominance hypothesis rather than the other two theories, since large contributions of overdominant genes and epistatic interactions have not been clearly identified yet. Inbreeding depression has been observed in several species and taxon [3, 4]. In cattle, inbreeding depression has been associated with reductions in productive traits, longevity and the ability of the individuals to cope with environmental challenges [5–8]. However, high levels of homozygosity have also been recently reported to be compatible with life and livelihood in cattle isolated for many generations (even up to 340 years) [9].

The increase in the average inbreeding coefficient observed in cattle during the last 20 years may have been caused by two main reasons: 1) the high selection intensity applied in the breeding schemes of highly productive breeds [10], and 2) the use of small and well-adapted populations in extensive and isolated production systems characterized by absence of breeding management [11]. The inbreeding coefficient F [12] is the most commonly used parameter in breeding schemes since it can be easily determined from pedigree data. Through its analysis, it has been suggested that the individual productivity begins to be affected when F reaches values higher than 5% [13]. For instance, Sewalem, Kistemaker [5] demonstrated that culling risk increased to near 14% when F values exceeded 6.25%. Therefore, individuals with F values higher than 20% are rarely found in commercial cattle herds [6], which constitutes the main source of genomic data destined to research in this species [14]. To our knowledge, the only existing study analyzing highly inbred livestock animals has been performed on a limited sample of Chillingham cattle (n = 16; [9]).

The F coefficient has been conventionally estimated from pedigree data (F_PED_) [15] or microsatellite markers (F_MIC_) [16]. However, the development of high-throughput genomic technologies based on SNP genotyping has allowed the establishment of novel approaches to determining inbreeding levels [17]. Nowadays, it has been demonstrated that SNP-based estimations of inbreeding are substantially more accurate and often less biased than F_PED_, even when large pedigrees are considered [18]. Additionally, SNP techniques allow the estimation of inbreeding values in individuals for which pedigree data are not available [19, 20]. One of the most common approaches for studying inbreeding consists in determining runs of homozygosity (ROH), which are long segments of the genome where the alleles are identical because both parents inherited them from a common ancestor (usually referred as autozygous) [21]. ROH analysis has been widely proposed and validated as a tool to estimate individual’s inbreeding level [14] in several species including beef [22–24] and dairy [25, 26] cattle. Moreover, the mean length of the ROH has been associated with the number of generations since the common ancestor [27], allowing to infer the demographic history of a given population.

Microarray density and parameter settings have been proven essential for accurate ROH identification [28]. In this aspect, proper settings (determined per Mb of ROH length) are crucial to avoid biased results due to genotyping errors and missing calls [14]. It has been demonstrated that medium-density (MD) genotype data tend to overestimate the number of fragments shorter than 4 Mb, since many heterozygous SNPs located within the fragments are not genotyped [28]. On the contrary, high-density (HD) microarrays provide a higher number of genotypes at the expense of more genotyping errors and missing calls, but these limitations can be accounted for by fine adjustment of software parameters, indicating that HD microarrays are a more reliable source of consistent results. Nevertheless, most studies in cattle have been performed using MD arrays (50,000 to 70,000 markers per individual), which is the way breeders associations normally genotype elite individuals for use in genomic-assisted breeding programs [25].

In humans, the accumulation of ROH in certain genomic positions has been used to analyze the demographic history of populations [29]. This strategy has also been employed to compare and characterize beef [22] and dairy cattle breeds [30]. Since ROH are normally abundant in regions under positive selection [31], their accumulation at specific loci or “hotspots” has been studied to identify genomic regions that reflect directional selection in cattle [22]. However, the same analysis has been recently employed to detect functional variants associated with inbreeding depression [32] and QTL´s [33]and the genetic control of reproductive traits [34, 35] and diseases [36]. On the other hand, regions with an unusually low abundance of ROH, (“cold spots” [37]) are thought to harbor loci with critical functions escaping lethal or damaging recessive variants. In both cases, it is worth mentioning that the inheritance of such autozygous fragments is also subject to the stochasticity of the recombination events across the genome. In this context, the accumulation of ROH along the genome has become the starting point for other techniques that aim at identifying biological factors acting behind the depressed phenotypes as alternatives to conventional GWAS studies [22, 33–36, 38].

Retinta is the second largest Spanish cattle breed characterized by the quality of its meat, its rusticity and its adaptation to marginal pasturelands and extreme weather [39]. All the individuals of this breed have been raised in the south part of the Spanish peninsula under dry and hot environmental conditions with scarce foraging resources. [40]. The Retinta cattle breeding plan was established more than three decades ago and has been focused on the selection of animals with increased growth speed and extended productive life, while maintaining its adaptation to harsh environments [41]. However, since inbreeding control is not often considered by breeders, this breed is a rare case of individuals with extremely variable F_PED_ coefficients within the population. This makes the Retinta breed an interesting model for animal inbreeding research.

The aims of this study were to characterize the occurrence of ROH in a Retinta population with extremely inbred and outbred animals. We analyzed the length, number, and genomic distribution of the ROH, obtained from HD genotyping data, as well as the relation between long ROH and recent inbreeding events retrieved from pedigree and microsatellite records. We also determined the differences among ROH patterns in a group of individuals with the same increase in F_PED_ values over the last 3 generations to evaluate the reliability of F_PED_ as a predictor of inbreeding depression. Finally, we estimated the influence of the recombination rate on the ROH patterns and identified loci putatively affected by inbreeding in highly related individuals through a functional annotation analysis.

## Materials and methods

### Animal samples

All the samples were obtained from the National Association of Breeders of Select Retinta Cattle (ACRE, by its Spanish acronym). DNA was obtained from frozen sperm straws (one per bull) using the HigherPurity™ Tissue Genomic DNA Purification Kit (Canvax Biotech, Cordoba, Spain). Management and treatment of the animals during sperm extraction were complied the ethical guidelines of the Animal Ethics Committee of the University of Cordoba, Spain. No ethics committee approval was necessary since no experimental procedures were carried out in animals during this study.

In total, we analyzed 54 bulls belonging to the artificial insemination (AI) program of the National Breeders Association of Select Retinta Cattle (ACRE, for its Spanish acronym). Individuals were selected based on the number of equivalent complete generations (ECG) and F_PED_ values. F_PED_, F_PED3_ (F_PED_ for last three generations) and ECG values were determined as described by Meuwissen and Luo [15] and Maignel, Boichard [42] using ENDOG software [43]. F_MIC_ was determined using a 17 STR-based genomic test recommended by the International Society for Animal Genetics for parentage testing as described by Caballero and Toro [44] using Molkin software [45]. For the purposes of our analyses, bulls were classified into two groups: inbred (HI, n = 32; average F_PED_ = 0.164; 0.103 to 0.306) and outbred (LI, n = 22; average F_PED_ = 0.008; 0 to 0.025).

### Genotyping

Samples were genotyped using the Axiom® Genome-Wide BOS 1 Bovine high-density SNP Array (Affymetrix, Santa Clara, Ca, USA) in a GeneTitan® Multi-Channel platform (Affymetrix). Samples were run at the IGEVET Genomics Core Facility (University of La Plata, La Plata, Argentina). Raw data were processed using Axiom™ Analysis Suite software (Affymetrix) and setting call rates and DQC at 0.97. Commonly used genotype filters such as minor allele frequency (MAF) were not applied since they would have led to an underestimation of ROH [28]. MAF filtering was avoided given the extreme inbreeding values existent in our population and the high number of SNPs with very low MAF. Discarding such a high number of SNPs would have interfered with some of the main purposes of our study Moreover, skipping such filters is common among ROH-related studies [9, 46]). SNPs that were either orphan or assigned to sex chromosomes or mitochondrial DNA were excluded from the analyses. The number of remaining variants was of 624,737.

### Detection and classification of ROH

ROH were estimated using cgaTOH software [47]. The minimum number of SNPs needed to constitute a ROH (L) was calculated according to Purfield, Berry [48], as follows:

$$L=\frac{\log_{e}(\frac{\alpha }{ns\times ni)})}{\log_{e}(1-het)}$$

where **n**_**s**_ is the number of SNPs per individual, **n**_**i**_ is the number of individuals, α is the percentage of false positive ROH (0.05), and **h**_**et**_ is the average SNP heterozygosity across all SNPs. ROH were divided into five different categories according to their length: 1–2 Mb, 2–4 Mb, 4–8 Mb, 8–16 Mb and >16 Mb. Short ROH (< 1 Mb) were not included in the analyses since many of them may derive from the inheritance of common allozygous haplotypes [25].

The number of heterozygous (**n**_**H**_) and missing (**n**_**M**_) genotypes allowed in each ROH category for each chromosome was calculated as

$$nH=\frac{mL}{dS}\times eG$$

nM=×mG

where **m**_**L**_ is ROH minimum length, **d**_**S**_ is the average distance between SNPs in the chromosome, **e**_**G**_ is the genotyping error rate (0.25% according to Affymetrix standard procedures), and **m**_**G**_ is the average missing genotype rate in the chromosome. The number of missing and heterozygous SNPs allowed per length category and chromosome are shown in S1 Table. Since each minimum length required a new round of ROH identification, fragments were merged in case of overlaps to avoid the underestimation of long ROH by using the *reduce* function of the *GenomicRanges* R package [49].

### ROH and ROH-based inbreeding coefficient (F_ROH)_ characterization

The mean, minimum and maximum length of the ROH and the standard error were determined for each sample group globally and also for each chromosome, individual and length category. Statistical differences were analyzed using a T standard test with a significance threshold of 0.05. F_ROH_ values were estimated for each individual and chromosome as the sum of all ROH divided by the genome (or chromosome) length, according to McQuillan, Leutenegger [21]. Results were expressed as Avg. ± standard error of the mean (S.E.M.)

We evaluated the reliability of F_ROH_ as inbreeding estimator compared with F_PED_, F_PED3,_ and F_MIC_ through Spearman correlations, since the distribution of the SNP data among samples was not normal (Kolmogorov–Smirnov test). The correlation between F_ROH>8Mb_ and F_PED_ (six generations)_,_ as well as between F_ROH>16Mb_ and F_PED3_ (three generations), were particularly estimated to evaluate the relationship between the length of ROH and the number of generations since the common ancestor, as was proposed by Fisher [50].

To determine if ROH were accumulated over loci with low recombination rates, we estimated the correlation between the number of ROH detected at a given position and the recombination rate at the same locus. To this end, recombination rate at each genomic position was obtained from data reported by Ma, O'Connell [51]. This was performed in the whole population and each group separately, both globally and by chromosome, and considering all ROH and ROH>8Mb.

### Gene clustering and functional analysis

To identify putative effects on the biological functions of the highly inbred animals, we performed a functional annotation analysis considering genes within loci that were statistically enriched with ROH. These loci were determined by performing a permutation test under the null hypothesis that ROH are equally distributed along the genome. The number of ROH at each position was randomized one million times using newly designed scripts in JULIA [52]. Then, the p-value for each position was calculated as the relative frequency of randomizations that produced a more extreme number of ROH than the one observed. Significantly enriched genomic intervals were defined as consecutive significant SNPs separated by no more than 1 Mb. These intervals were used to retrieve gene lists from ENSEMBL BioMart v89 [53]. The in silico analysis was conducted using the Functional Annotation Clustering tool implemented in DAVID [54] considering the following annotation categories: *Cog_Ontology*, *Up_Keywords*, *Up_Seq_Feature*, *Goterm_Bp_Direct* (biological processes), *Goterm_Cc_Direct* (cellular components), *Goterm_Mf_Direct* (molecular functions), *Kegg_Pathway*, *Interpro*, and *Smart*. The classification stringency was set to medium and groups were defined by enrichment scores greater than 1.301, which is equal to–log_10_ (0.05). The analysis was performed on the full set of statistically enriched regions (considering all ROH, regardless of their length) and on a reduced set comprising only ROH>8Mb to evaluate the effect of recently formed fragments.

## Results

### ROH characterization

The number of ROH was statistically different between the HI and LI groups (106.96 ± 37.56 in HI vs 39.63 ± 23.67 in LI; P<0.001). This statistical difference was also observed at the length category level (Table 1). The HI/LI relation in ROH>8Mb is more than four times higher than in short ROH, demonstrating a clearly higher occurrence of long ROH in the inbred group.

**Table 1:** Number of ROH detected by length category. Differences between groups were analyzed using a T-test. Results are expressed as mean ± S.E.M.

ROH Lenght	Group HI			Group LI			P-Value	HI/LI
1–2 Mb	61.13	±	5.20	28.91	±	3.85	3.00E-05	2.11
2–4 Mb	22.28	±	1.71	7.00	±	1.15	2.08E-08	3.18
4–8 Mb	12.56	±	1.12	2.50	±	0.56	9.90E-09	5.03
8–16 Mb	7.47	±	0.89	0.86	±	0.25	1.56E-07	8.65
> 16 Mb	3.53	±	0.55	0.36	±	0.14	2.10E-05	9.71

**Fig 1:**
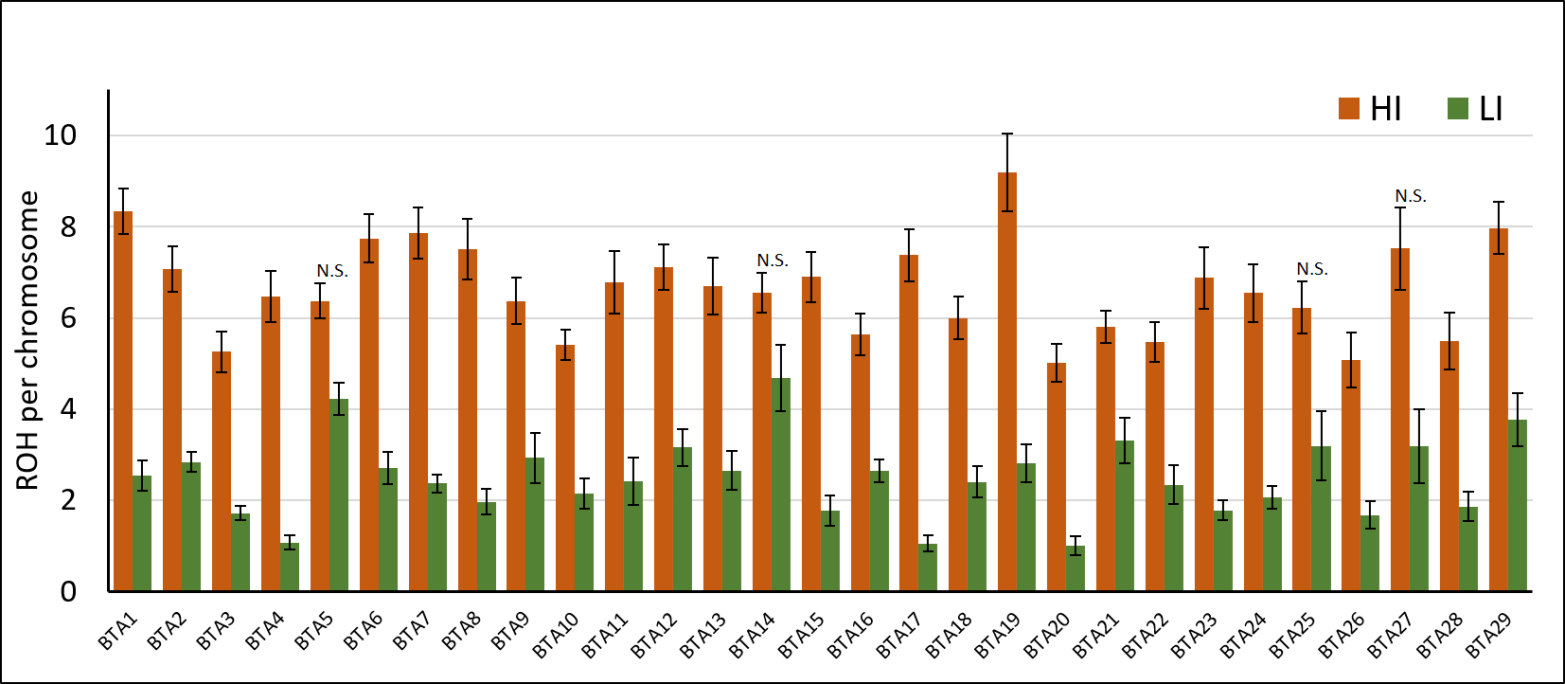
Number of ROH by chromosome in inbred (HI) and outbred (LI) bulls. Results are expressed as average number of ROH per chromosome ± S.E.M. N.S.: Not significant; P>0.05; T-test.

A similar analysis performed by chromosome showed similar results, with statistical differences in 25 different chromosomes (BTA5, BTA14, BTA25, and BTA27 were not significant; P>0.05; Fig 1), suggesting that some chromosomes are more propense to accumulate ROH than others.

**Fig 2:**
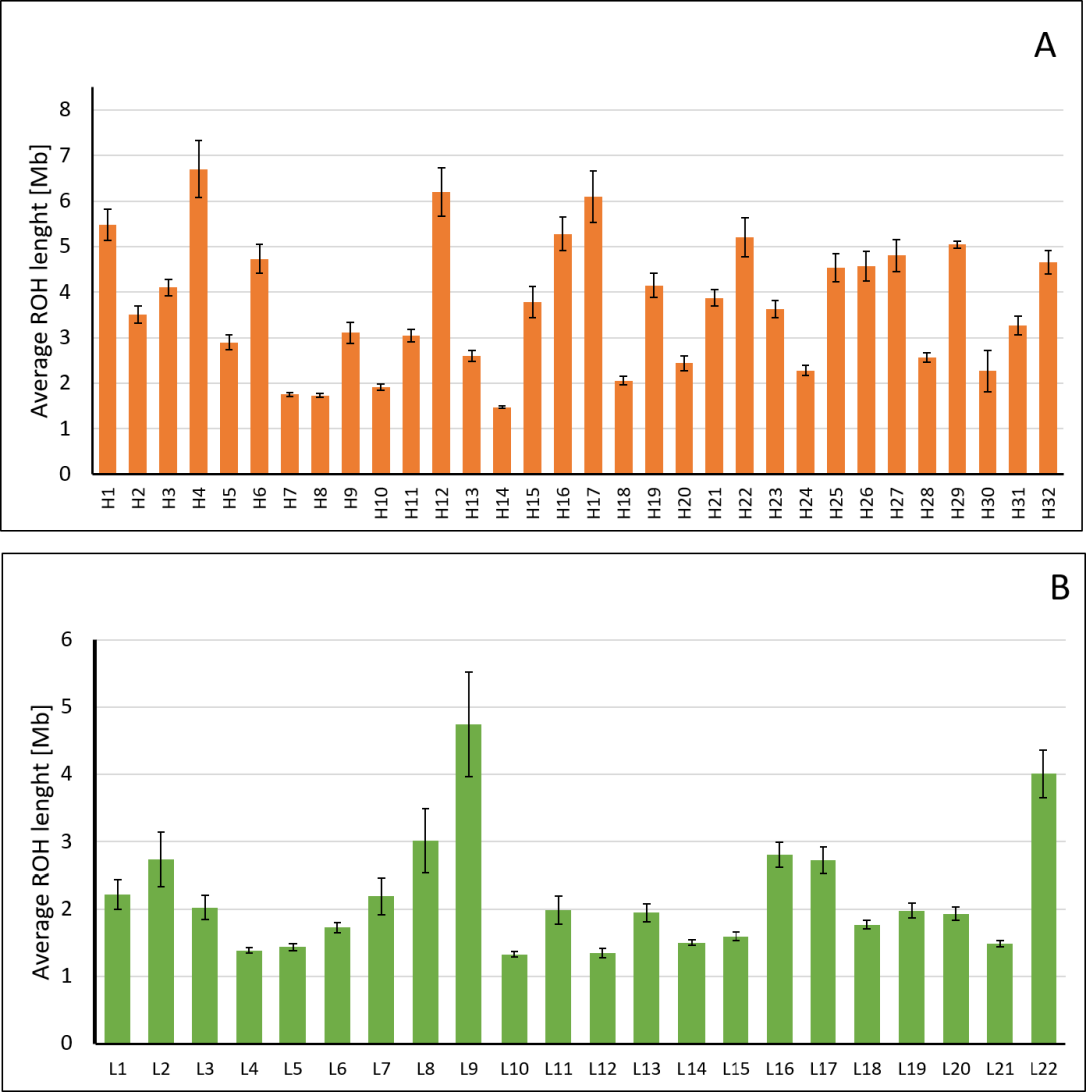
Individual variation in ROH length in inbred (HI; 2A) and outbred (LI; 2B) bulls. Results are expressed as average length ± S.E.M. by individual.

The average ROH length was also significantly different between groups (3.51 ± 0.08 Mb in HI vs 2.23 ± 0.10 Mb in LI; P < 0.001) (Fig 2A), but HI showed a higher variation among individuals (Fig 2B). In this sense, maximum length varied from 4.71 (H24) to 66.78 Mb (H4). Samples H7, H8, H10, H14, H24, and H30 showed a lower variability, lacking ROH > 11.5 Mb even though their F_PED3_ values were higher than 0.10. The LI group showed a lower variation, although samples L2, L8, L9 and L22 seemed slightly more variable in length of ROH (Fig 2B). Samples L2, L8, L9, L16, L17, and L22 showed ROH>16 Mb even though their F_PED_ was of zero.

**Fig 3:**
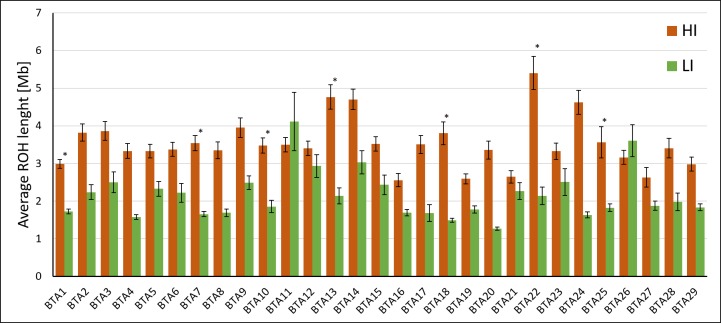
Chromosomal variation in ROH length in inbred (HI) and outbred (LI) bulls. Results are expressed as average length ± S.E.M. by individual. *: P < 0.05 T-test by group.

Significant differences were also observed in the length of ROH by chromosome but in a lesser extent. Only seven chromosomes showed statistical differences between groups (BTA1, BTA7, BTA10, BTA13, BTA18, BTA22, BTA24; P < 0.05; Fig 3). The number of chromosomes containing ROH longer than 20 Mb was higher in HI (25 chromosomes) than in LI (6 chromosomes). Surprisingly, a very long ROH (56.73 Mb) was detected on BTA11 in the L9 sample. Details on the distribution and length of ROH by individual and chromosome are available in S2 and S3 Tables, respectively.

### Comparison of F_PED_, F_MIC_, and F_ROH_ as identity by descent (IBD) estimators

The comparison between IBD estimators showed significant differences for all the inbreeding estimators, but for F_MIC_, the p-value was at least two orders higher (Table 2). Additionally, F_MIC_ showed the highest value in the LI group as well as the lowest HI/LI ratio, suggesting that STR-based inbreeding coefficients may tend to overestimate the value in outbred individuals.

**Table 2:** Pedigree-based (FPED), ROH-based (FROH) and microsatellite-based (FMIC) inbreeding coefficients for highly inbred (HI) and outbred (LI) bulls.

	HI			LI			P-Value	HI / LI
F_PED_	0.1644	±	0.0101	0.0080	±	0.0001	1.00E-06	20.68
F_PED3_	0.0830	±	0.0121	0			-----	-----
F_MIC_	0.1595	±	0.0613	0.1155	±	0.0001	1.46E-02	1.38
F_ROH_	0.1510	±	0.0091	0.0356	±	0.0059	5.40E-06	4.24
F_ROH[__1__–__2__]_	0.0320	±	0.0027	0.0150	±	0.0037	1.10E-05	2.14
F_ROH[__2__–__4__]_	0.0247	±	0.0018	0.0077	±	0.0001	1.30E-06	3.20
F_ROH[__4__–__8__]_	0.0272	±	0.0025	0.0054	±	0.0001	4.20E-05	5.07
F_ROH[__8__–__16__]_	0.0317	±	0.0039	0.0038	±	0.0001	1.00E-06	8.26
F_ROH[>16]_	0.0354	±	0.0057	0.0037	±	0.0001	6.10E-06	9.55

HI/LI: fold increase between high and low inbreeding. P-values calculated through independent T-test.

In the HI group, F_ROH_ values ranged from 0.06 to 0.28 (0.15 ± 0.09), showing a high correlation with F_PED_ (0.79). On the contrary, correlations between F_PED_ and F_MIC_ (0.44) and F_ROH_ and F_MIC_ (0.36) were much lower, which is consistent with comparisons performed between groups. Correlations between F_PED_ and F_ROH>8Mb_, F_PED3_ and F_ROH>16Mb_, and F_PED3>0_ (individuals with a common ancestor in the last three generations) and F_ROH>16Mb_ were 0.60, 0.54 and 0.42, respectively.

### Analysis of F_ROH_ estimates by fragment length and chromosome

**Fig 4:**
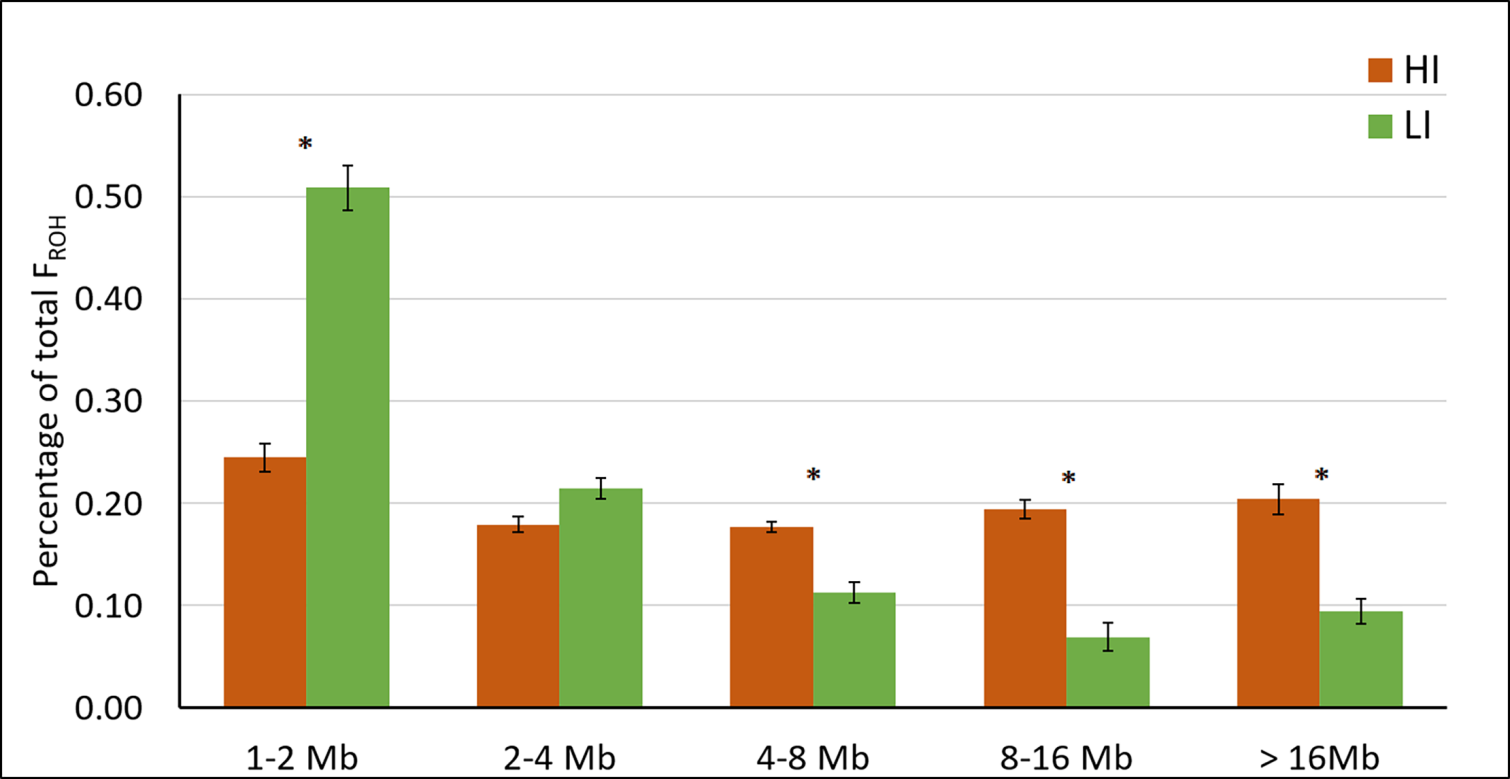
Percentage of F_ROH_ value explained by each fragment length category. *: P<0.05. T-tests by group.

F_ROH_ values were significantly higher in the HI group (P<0.001). The percentage of F_ROH_ value explained by each length category is shown in Fig 4. Short ROH (1–2 Mb) accounted for nearly 50% of the F_ROH_ value in LI, showing a decrease in contribution towards the 8–16 Mb category. On the other hand, a similar contribution was observed for each length category in HI. The percentage of F_ROH_ explained by short fragments (1–2 Mb) was significantly higher in LI, whereas the percentage explained by long fragments (4–8 Mb, 8–16 Mb, and >16 Mb) was significantly higher in HI.

**Fig 5:**
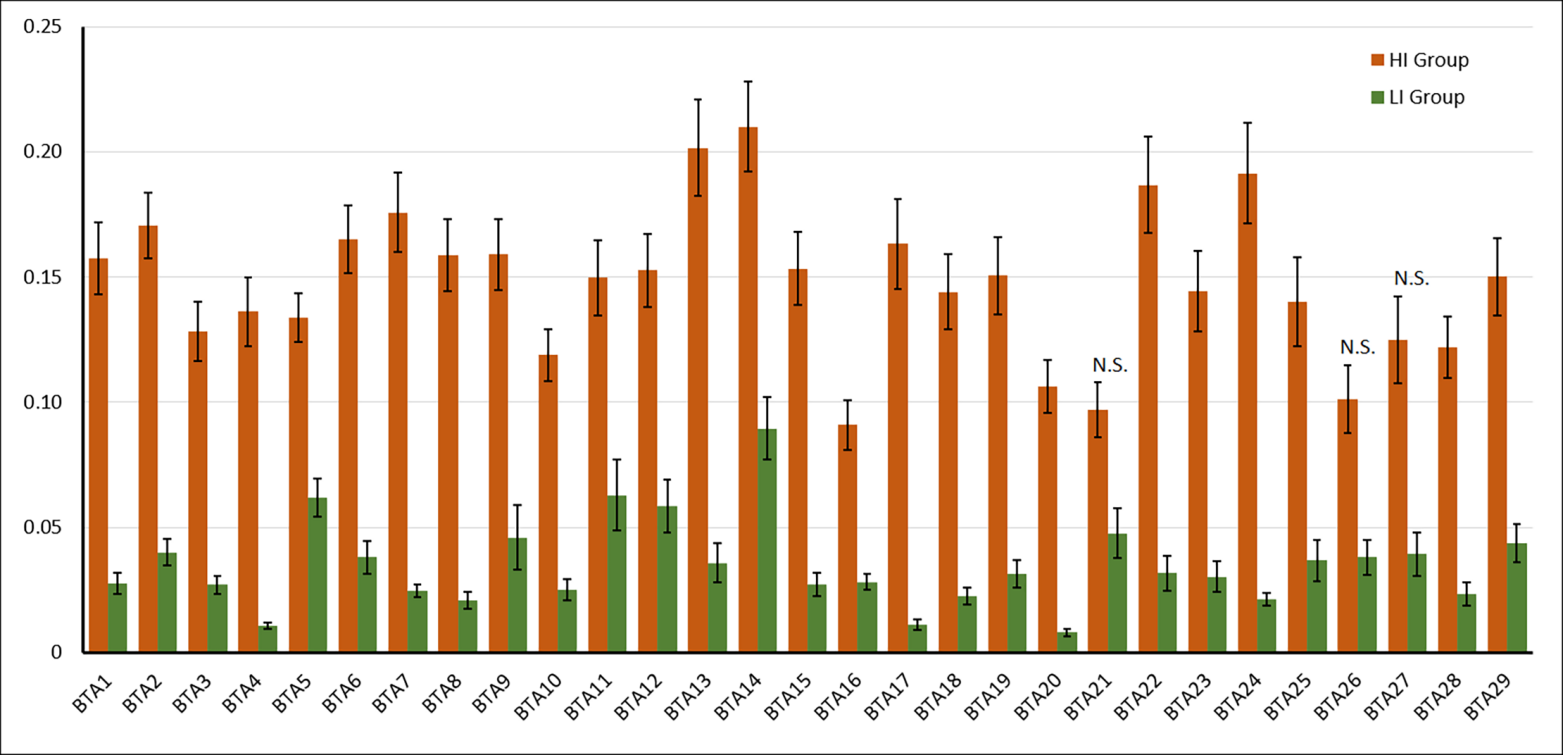
FROH by chromosome in highly inbred bulls (HI) and outbred bulls (LI). Only chromosomes marked as N.S. were nonsignificant (P > 0.05).

The analysis by chromosome showed a higher F_ROH_ in HI in 26 chromosomes (Fig 5; P<0.05). Only BTA21, BTA26, and BTA27 were not significant, probably due to the variability observed among individuals, rather than by a decreased difference between means. The variability within groups was high, but some chromosomes, such as BTA14, showed a higher abundance of ROH than others (e.g. BTA20) in both groups. It is also noteworthy that some outbred individuals showed unexpectedly high abundance in some of the chromosomes. For example, sample L9 had 57% of chromosome BTA11 covered by a single ROH (data not shown).

### Effect of the recombination rate on ROH abundance

The correlation between the occurrence of a ROH at a given position (defined as the number of individuals with a ROH at that position) and the recombination rate at the same locus was -0.0132. Similar results were obtained when the analysis was performed by group (r = -0.0178 in HI and r = -0.02715 in LI) and chromosome (S4 Table). When all samples from both groups were analyzed at the same time, the highest correlation was found at BTA23 (r = 0.11). BTA28 showed the highest correlation in HI (r = 0.11) and BTA26 the highest one in LI (0.08). No strong correlations were either observed in the analysis of putative recent ROH (r = 0.0025; 0.0003 and 0.028 for the full sample set, HI and LI respectively) nor in the case of individual chromosomes (S4 Table).

### Genomic localization of ROH´s

**Fig 6:**
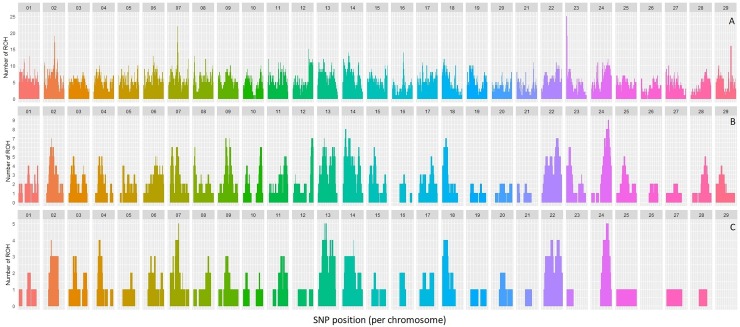
Accumulation of ROH across the genome. Number of ROH detected at each SNP position of the array considering ROH of different length categories. Footnote: A: Total F_ROH_; B: 8–16 MB F_ROH_; C: >16 Mb F_ROH_.

The accumulation of ROH across the genome of the highly inbred samples was analyzed by length category: all ROH lengths (Fig 6A), ROH>8Mb (Fig 6B) and ROH>16Mb (Fig 6C). Although the distribution of the ROH was relatively even and accumulation was moderate in general, we found a few outstanding peaks with a high occurrence of ROH. For example, 26 animals presented ROH at the first million bases (1,575–1,606,000) of BTA23, as well as in BTA2 (71,882,569–73,223,813), BTA7 (51,003,562–53,466,651) and BTA29 (38,490,349–39,617,454) to a lower extent. In the analysis of ROH > 8 Mb (hypothetically generated during the last six generations), the highest occurrence (nine individuals) was detected at BTA24 (39,376,427–58,300,844). Lastly, when we analyzed ROH>16Mb (three generations since the common ancestor), the highest occurrence was found at BTA 7, BTA13, BTA14, BTA18, BTA22 and BTA24. Interestingly, two chromosomes (BTA26 and BTA29) showed a complete absence of ROH> 16Mb.

**Fig 7:**
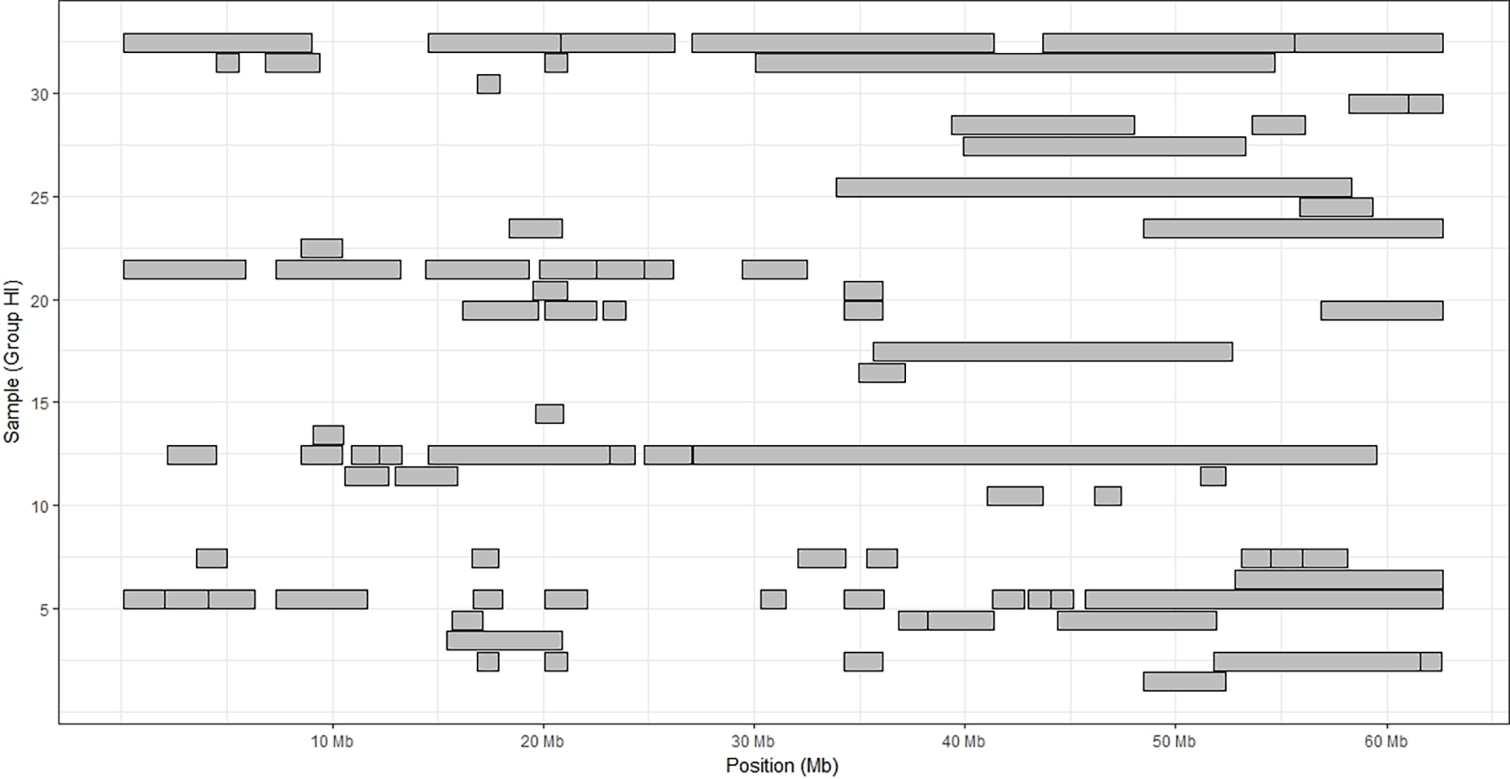
Physical distribution of ROH over chromosome BTA24 in the HI group.

To further show the high variability within chromosomes, we illustrated the distribution of the ROH on BTA24, which showed the highest average F_ROH_ and average length of ROH in the HI group (Fig 7). It is noteworthy that some individuals showed no ROH across all the chromosome (H1, H9, H10, H15, and H26). On the contrary, some individuals harbored ROH in more than 60% of their chromosomal extension (H13 and H32).

**Fig 8:**
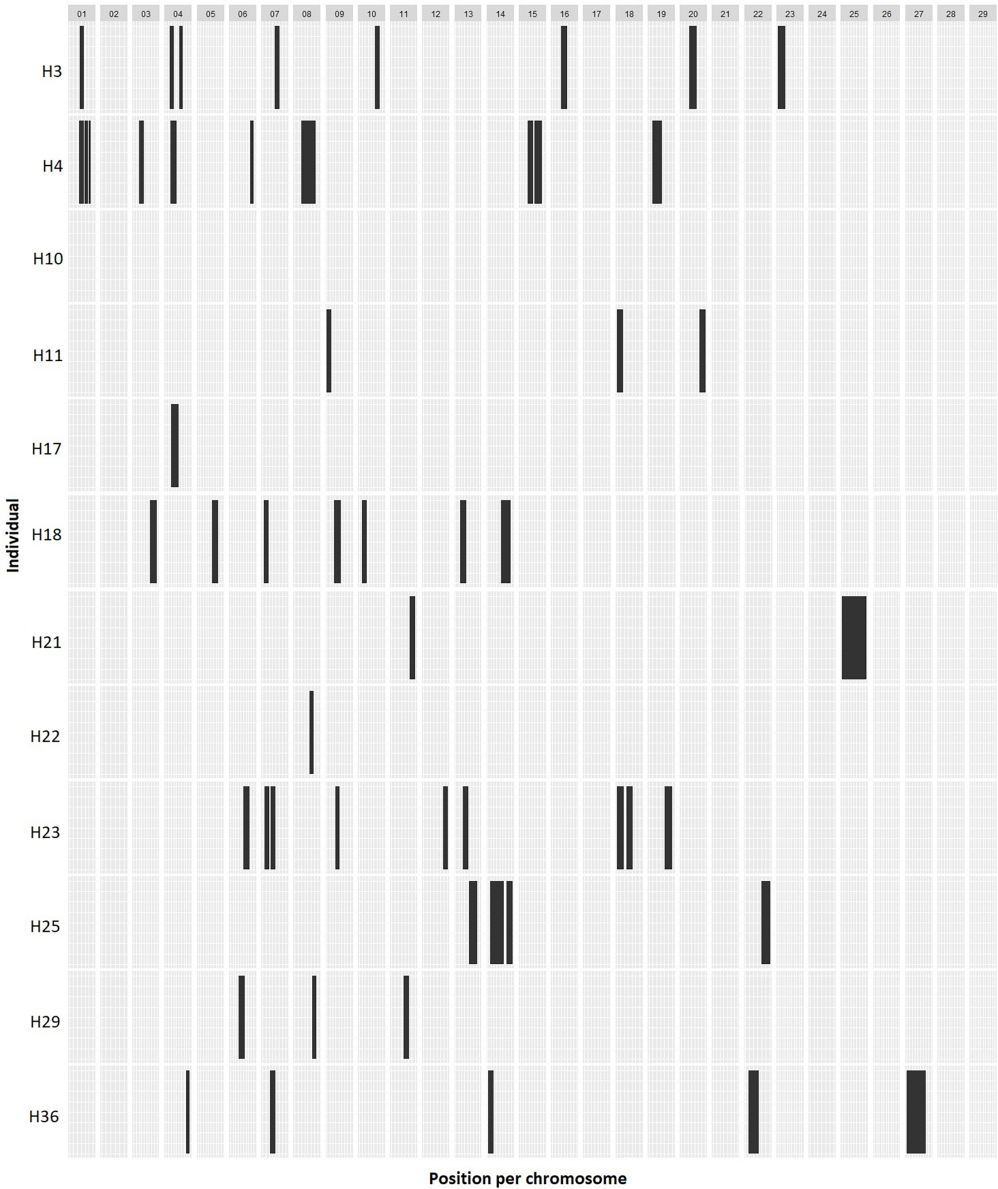
Physical distribution of ROH>16Mb in individuals with the same inbreeding coefficient during the last three generations (F_PED3_ = 0.125).

Finally, the relation of F_PED_ with IBD was analyzed by plotting the genome-wide distribution of ROH>16Mb in 12 individuals with identical F_PED3_ (0.125) (Fig 8). The pattern showed that the distribution of IBD fragments hypothetically acquired during the last 3 generations was highly uneven and variable among individuals. Furthermore, no ROH>16Mb were detected in the H10 sample nor in seven chromosomes (BTA2, BTA17, BTA21, BTA24, BTA26, BTA28 and BTA29). These results suggest that the phenotypic effect of inbreeding in individuals with the same F_PED_ value could be highly divergent.

### Candidate regions and genes

To analyze putative biological functions affected in zones with high ROH abundance, we retrieved a list of genes from the intervals significantly enriched based on the permutation test. As before, to characterize recently formed fragments, we conducted separate analysis for all ROH fragments (no length restriction) and for ROH>8Mb. The lists of genes obtained were then submitted to DAVID Bioinformatics for functional annotation clustering. When all fragment lengths were considered, the number of retrieved known genes was of 769. These genes were distributed across 22 different chromosomes, but eight of them were lightly represented (less than 10 genes). The most represented chromosomes in terms of genes covered by ROH were BTA7, BTA14, and BTA12 with 108, 94 and 77 genes, respectively. Three significant functional clusters were detected in the annotation clustering analysis (Table 3). The one with the highest enrichment score was related to protein catabolic processes and involved the PAG family of genes. These genes are located on BTA29 and code for precursors of the pregnancy-associated glycoprotein family (PAG1, PAG4, PAG7, PAG15, PAG16, PAG19, PAG20, and PAG21), a group of aspartic proteases secreted by the placenta. The second most enriched cluster was related to 5 β-defensin genes, which codify for antimicrobial peptides found in white blood cells.

**Table 3:** Functional annotation clustering of genes in regions with high ROH accumulation.

**Cluster 1**	**Enrichment Score**: 8.96			
Category	Term	P Value	Genes	Fold Enrichment
*INTERPRO*	Propeptide, peptidase A1 (IPR012848)	2.45E-13	*PAG1*, *PAG4*, *PAG5*, *PAG7*, *PAG15*, *PAG16*, *PAG17*, *PAG18*, *PAG19*, *PAG20*,	14.36
			*PAG21*, *LOC504812*, *LOC614287*, *MGC157405*, *MGC157408*	
*INTERPRO*	Peptidase A1 (IPR001461)	1.35E-12	*PAG1*, *PAG4*, *PAG5*, *PAG7*, *PAG15*, *PAG16*, *PAG17*, *PAG18*, *PAG19*, *PAG20*,	13.02
			*PAG21*, *LOC504812*, *LOC614287*, *MGC157405*, *MGC157408*	
*INTERPRO*	Aspartic peptidase (IPR021109)	1.48E-11	*PAG1*, *PAG4*, *PAG5*, *PAG7*, *PAG15*, *PAG16*, *PAG17*, *PAG18*, *PAG19*, *PAG20*,	11.26
			*PAG21*, *LOC504812*, *LOC614287*, *MGC157405*, *MGC157408*	
*GOTERM_MF_DIRECT*	Aspartic-type endopeptidase activity (GO:0004190)	1.22E-10	*PAG1*, *PAG4*, *PAG5*, *PAG7*, *PAG15*, *PAG16*, *PAG17*, *PAG18*, *PAG19*, *PAG20*,	9.76
			*PAG21*, *LOC504812*, *LOC614287*, *MGC157405*, *MGC157408*	
*INTERPRO*	Peptidase aspartic, active site (IPR001969)	2.00E-10	*PAG1*, *PAG4*, *PAG5*, *PAG7*, *PAG15*, *PAG16*, *PAG17*, *PAG18*, *PAG20*,	12.03
			*PAG21*, *LOC504812*, *LOC614287*, *MGC157405*	
*UP_KEYWORDS*	Aspartyl protease	2.08E-10	*PAG1*, *PAG4*, *PAG5*, *PAG7*, *PAG15*, *PAG16*, *PAG17*, *PAG18*, *PAG20*,	12.00
			*PAG21*, *LOC504812*, *LOC614287*, *MGC157405*	
*GOTERM_BP_DIRECT*	Protein catabolic process (GO:0030163)	2.15E-09	*PAG1*, *PAG4*, *PAG7*, *PAG15*, *PAG16*, *PAG17*, *PAG18*, *PAG20*,	8.97
			*PAG21*, *LOC504812*, *LOC614287*, *MGC157405*	
*GOTERM_BP_DIRECT*	Proteolysis (GO:0006508)	0.0002	*GGH*, *PAG1*, *PAG4*, *PAG5*, *PAG7*, *PAG15*, *PAG16*, *PAG17*, *PAG18*, *PAG20*,	2.83
			*PAG21*, *LOC504812*, *LOC614287*, *MGC157405*, *PROZ*, *TPP2*	
*UP_KEYWORDS*	Protease	0.0002	*CASP8*, *CLPP*, *CTSB*, *CTSC*, *CTSZ*, *ECE2*, *F7*, *F10*, *PAG1*, *PAG4*, *PAG5*, *PAG7*, *PAG15*, *PAG16*, *PAG17*, *PAG18*,	2.27
* *			*PAG19*, *PAG20*, *PAG21*, *PARL*, *NPEPL1*, *USP4*, *USP14*, *TPP2*, *LOC504812*, *LOC614287*, *MGC157405*	
**Cluster 2**	**Enrichment Score: 2.03**			
Category	Term	P Value	Genes	Fold Enrichment
*SMART*	*DEFSN (SM00048)*	0.0005	*DEFB1*, *DEFB4A*, *DEFB5B*, *DEFB10*, *EBD*	12.18
*INTERPRO*	*Beta defensin/Neutrophil defensin (IPR006080)*	0.0006	*DEFB1*, *DEFB4A*, *DEFB5B*, *DEFB10*, *EBD*	11.57
*INTERPRO*	*Beta defensin type (IPR001855)*	0.0012	*DEFB1*, *DEFB4A*, *DEFB5B*, *DEFB10*, *EBD*	9.92
*UP_KEYWORDS*	*Defensin*	0.0061	*DEFB1*, *DEFB4A*, *DEFB5B*, *DEFB10*, *EBD*	6.59
*UP_KEYWORDS*	*Antibiotic*	0.0204	*DEFB1*, *DEFB4A*, *DEFB5B*, *DEFB10*, *EBD*, *PENK*	3.78
*UP_KEYWORDS*	*Antimicrobial*	0.0445	*DEFB1*, *DEFB4A*, *DEFB5B*, *DEFB10*, *EBD*, *PENK*	3.08
GOTERM_BP_DIRECT	*Defense response to bacterium (GO*:*0042742)*	0.073291513	*DEFB1*, *DEFB4A*, *DEFB5B*, *DEFB10*, *EBD*, *NOS2*, *PENK*	2.38
**Annotation Cluster 3**	**Enrichment Score:** 1.54			
Category	Term	P Value	Genes	Fold Enrichment
*UP_KEYWORDS*	*Microtubule*	0.0052	*AURKA*, *DYNLL2*, *DYNLRB2*, *HAUS1*, *KATNAL2*, *KIF20A*, *NDRG1*, *REEP4*, *SKA1*,	2.68
			*TUBB1*, *TUBB4A*, *TUBGCP3*	
*UP_KEYWORDS*	*Cytoskeleton*	0.0485	*AURKA*, *CETN1*, *CETNNA1*, *DAG1*, *DMTN*, *DYNLL2*, *DYNLRB2*, *FAM110B*, *FER*, *HAUS1*, *KATNAL2*,	1.55
			*KIF20A*, *MAP6D1*, *NDRG1*, *PDLIM2*, *PPP2CA*, *REEP4*, *RHOA*, *ROCK1*, *SKA1*, *TUBB1*, *TUBB4A*, *TUBGCP3*	
*GOTERM_CC_DIRECT*	*Microtubule (GO*:*0005874)*	0.0968	*DYNLL2*, *DYNLRB2*, *HAUS1*, *KATNAL2*, *KIF20A*, *NDRG1*, *REEP4*, *SKA1*, *MAP6D1*	1.83
* *			*TUBB1*, *TUBB4A*	

When focused on regions ROH>8Mb, that hypothetically formed during the last six generations, we retrieved 362 known genes. These genes were distributed over seven chromosomes. BTA22, BTA24, and BTA14 were the most represented ones, with 87, 68 and 64 genes, respectively. Three significant clusters were identified in the functional annotation analysis (Table 4). The most significant one was related to the assembly of the flagellum and cilia microtubules involving 4 genes (*DNAAF1*, *DNAH1*, *LRRC6*, and *ZMYND10*). The remaining two clusters were related to calcium binding and metabolism, and hyaluronan metabolism.

**Table 4:** Functional annotation clustering of genes in regions with high accumulation of putative modern ROH (>8Mb).

**Cluster 1**	**Enrichment Score**: 2.265			
Category	Term	P Value	Genes	Fold Enrichment
*GOTERM_BP_DIRECT*	Inner dynein arm assembly (GO:0036159)	0.0005	ZMYND10, LRRC6, DNAH1, DNAAF1	23.23361345
*GOTERM_BP_DIRECT*	Motile cilium assembly (GO:0044458)	0.0145	ZMYND10, LRRC6, DNAAF1	15.84110008
*GOTERM_BP_DIRECT*	Outer dynein arm assembly (GO:0036158)	0.0202	ZMYND10, LRRC6, DNAAF1	13.40400776
**Cluster 2**	**Enrichment Score:** 1.825			
Category	Term	P Value	Genes	Fold Enrichment
*SMART*	IQ motif (SM00015)	0.0071	IQCF1, LOC100125949, IQCF2, IQCF5, MYOB5B	6.456855792
*UP_SEQ_FEATURE*	IQ 1 (domain)	0.0085	IQCF1, IQCF2, IQCF5	20.43586957
*UP_SEQ_FEATURE*	IQ 2 (domain)	0.0085	IQCF1, IQCF2, IQCF5	20.43586957
GOTERM_MF_DIRECT	Calmodulin binding (GO:0005516)	0.0322	MAPKAPK3, IQCF1, IQCF2, KCNQ3, IQCF5	4.140842398
*INTERPRO*	IQ motif, EF-hand binding site (IPR000048)	0.0448	IQCF1, IQCF2, IQCF5, MYOB5B, LOC100125949	3.731290251
**Cluster 3**	**Enrichment Score:** 1.596			
Category	Term	P Value	Genes	Fold Enrichment
*GOTERM_BP_DIRECT*	Hyaluronan metabolic process (GO:0030212)	0.0001	ITIH3, HYAL1, ITIH1, ITIH4	38.72268908
*UP_SEQ_FEATURE*	VIT (domain)	0.0019	ITIH3, ITIH1, ITIH4	40.87173913
*INTERPRO*	Inter-alpha-trypsin inhibitor heavy chain, C-terminal (IPR010600)	0.0028	ITIH3, ITIH1, ITIH4	35.37263158
*SMART*	VIT (SM00609)	0.0053	ITIH3, ITIH1, ITIH4	26.01190476
*INTERPRO*	VIT domain (IPR013694)	0.0095	ITIH3, ITIH1, ITIH4	19.65146199
*UP_SEQ_FEATURE*	VWFA (domain)	0.0415	ITIH3, ITIH1, ITIH4	9.082608696

## Discussion

In this study, we characterized the abundance and distribution of ROH in inbred and outbred cattle. To our knowledge, this is the first genomic report to use HD genotyping to analyze the relation between IBD fragments and known recent inbreeding events in individual cattle with extreme F values. This is also the first analysis of a cattle population that has been bred and selected under standardized conditions and can be clustered into two highly divergent groups in terms of individual inbreeding values.

### F_ROH_ as inbreeding estimator on highly inbred cattle

Our results showed a higher correlation between F_PED_ and F_ROH_ in comparison with previous reports [25], but only when the whole set of samples was considered. Correlations were lower when both groups were analyzed separately, showing values more consistent with those reported previously [14]. Three causes were suggested as source of divergence between F_ROH_ and F_PED_: 1) the persistence of ancestral short ROH through time due to low recombination rates, which are ignored in the estimation of F_PED_ [55], 2) the depth and reliability of pedigree information [18, 56] and 3) the stochastic nature of IBD inheritance [57]. In this study, pedigree errors were minimized using molecular parentage tests for all individuals. Similarly, pedigree data were shown reliable since outbred animals (LI) showed low F_ROH_ values, which may indicate that inbreeding events that occurred in the population before the fifth parental generation are scarce. For this reason, our correlations are hypothetically reliable. On the contrary, the STR-based inbreeding estimations (F_MIC_) were poorly correlated with both F_PED_ and F_ROH_, demonstrating that the use of STR is a poor source of information in highly inbred individuals. This fact was previously described in cattle but using a moderately inbred population, as was shown by Baumung and Solkner [58].

Seven years ago, Howrigan, Simonson [27] modeled the relationship between the length of ROH and the number of generations since the common ancestor by large-scale simulations. The study was based on the concept proposed by Fisher [50] in which, the length of the IBD fragments is associated with the number of generations since the common ancestor. For instance, they showed that ROH>16Mb are likely inherited from parents that shared a common ancestor three generations before. More recently, Marras, Gaspa [24] and Ferencakovic, Hamzic [55] analyzed this concept in commercial populations of several cattle breeds but using individuals with low F_PED_. Since our population had a well-known history of recent inbreeding, we expected a higher correlation between estimates coming from long ROH (F_ROH_ derived from ROH>8 and ROH>16) and F_PED_ values from the corresponding number of generations (F_PED_ for six and F_PED3_ for three). There was a general agreement between increased ROH lengths and inbreeding events in the inbred individuals, showing correlations between r = 0.6 and r = 0.42 when the long ROH fragments were used to estimate F_ROH_. However, these correlations were lower than the overall correlation obtained using the analysis of the whole population. One possible explanation is the stochastic nature of IBD inheritance [57]. However, Kardos, Luikart [18] demonstrated that F_PED_ can be easily underestimated when the pedigree depth is shorter than 20 generations. In our case, F_PED_ was estimated using only 6 generations on average, thus the influence of previous common ancestors was not accounted for.

### ROH length distribution

The length of the ROH was highly variable between and within the two sample groups. For instance, five LI samples showed ROH longer than 20 Mb, when presence of long ROH was not expected. Although the mean length of the ROH should at some extent reflect the number of generations since the common ancestor, we should also consider that ROH formation is a complex process involving dynamic rates of recombination along the genome and the stochastic nature of gamete formation processes [38]. In our study, this hypothesis is also supported by the variation detected in the HI group, where some animals had ROH as long as 50 Mb (produced hypothetically by an inbreeding event that occurred one generation ago), which was inconsistent with the F_PED_. A similar variability was also described in Brown Swiss cattle and Valle de Belice sheep [28, 38]. However, in both studies pedigree data was not available, thus preventing the comparison between F_PED_ and ROH length.

Significant differences were also observed in the number of ROH detected by length category. Shorter ROH predominated in LI, but the number of ROH decreased as the length increased. This pattern may be attributed to a “foundational inbreeding” produced during the creation of the breed, as suggested in other breeds and species [22, 24, 59]. On the other hand, HI showed a number of ROH [8-16Mb] and ROH>16 Mb that was eight and ten times higher than in LI. To our knowledge, high differences in the number of ROH were not reported previously in livestock. In our case, the experimental design, including two groups of animals which diverged only in terms of inbreeding (same breed, selection scheme, and breeding objectives) could explain the differences observed between groups. In any case, our results were consistent with expectations, since inbreeding events that occurred more recently (6 generations) tended to produce longer ROH fragments. The fact that only 1.5% percent of the genome was covered by short ROH in LI may indicate that the original population from which the Retinta breed was founded 50 generations ago was ample and scarcely selected.

### Chromosomal distribution of ROH

In a recent study, Zavarez, Utsunomiya [59] reported a reduced variation in the distribution of the ROH among the chromosomes of Nelore cows, with FROH values ranging from 0.05 to 0.1. On the contrary, Ferencakovic, Solkner [28] and Szmatoła, Gurgul [22] showed that the length of the ROH and their localization in the genome could be extremely variable in cattle. In our case, the degree of variation among chromosomes was high, which agrees with the latter. This variation within and between chromosomes has been also described in dairy [56] and beef [22] cattle populations, but with low inbreeding levels and with a lesser extent. These findings may suggest that some genomic loci are less able to sustain accumulation of IBD than others. In our case, it may be explained by the existence of deleterious alleles located in ROH located in specific regions (For instance in regions with systematically lower genomic constraint [60]). Those alleles will decrease the ROH abundance in those locations since if a variant in a population is lethal in homozygous form, inbreeding will greatly increase the chance of generating a lethal genotype. In this experiment, it is reasonable to think that the hotspots detected might have been produced mostly because of mating close relatives in absence of additional conditions that can differentiate the groups analyzed. But also, the fact that recombination rates, which have been also pointed as the cause of ROH increase in specific regions, seemed to exert no effects on ROH abundance, is supporting the previous idea. Finally, variation was also high when we analyzed the genome distribution of ROH>8Mb and ROH>16Mb. Interestingly, some genomic regions were found to lack long ROH (e.g. BTA26 and BTA29 for ROH>16Mb). These findings may suggest that some genomic areas cannot be affected by inbreeding within a short time lapse.

The uneven genome-wide distribution of the ROH is particularly important since breeders usually associate an increase in the inbreeding coefficient with a proportional detrimental effect [61, 62]. However, the phenotypic effects of the IBD blocks will be determined by their localization on the genome. Forty years ago, Franklin [63] modeled and determined variation expected in terms of homozygous blocks in individuals resultant from a similar inbred event in *D*. *melanogaster*. In that study, the author demonstrated that the chance of finding a homozygous genotype at a specific position of the genome after an event of inbreeding depends on the recombination rate of the locus and the length of the chromosome. According to the author, the abundance of inbreeding blocks should be higher in chromosomes longer than 1M, but the variability should be lower in chromosomes shorter than 0.5M. To show that, we analyzed the distribution of recently formed ROH (>8Mb and >16Mb) on 12 individuals with the same increase in F during the last three generations (F_PED3_ = 0.125). Once again, results showed an uneven genome distribution with a moderate accumulation of ROH over specific loci (up to nine ROH>8Mb on some loci of BTA24). Besides, seven chromosomes showed a total absence of ROH >16Mb. Despite the limited number of samples, our study shows that high inbreeding levels might not always lead to inbreeding depression, as was recently also shown in a study of isolated cattle [9]. In fact, even the mating of unrelated animals might possibly lead to inbreeding depression if, by chance, they happened to present long ROH at some biologically important loci. We showed that although accumulation of fragments over specific loci is not common, such long ROH are easy to find among outbred individuals. Furthermore, we showed that the genome-wide distribution of the ROH was generally uneven, which means that the phenotypic effects of inbreeding depression may vary between animals regardless of their F_PED_.

### Functional annotation clustering

The functional study of genomic regions significantly enriched with ROH constitutes a practical approach to identify metabolic pathways putatively affected by inbreeding. In our case, we performed two separate analyses: one considering all the ROH fragments and another one considering only putatively modern ROH. The purpose of this separation was to distinguish the putative effects of short-term inbreeding from the rest. But also, it was suggested that the analysis of biological functions affected by recent inbreeding (ROH >8 Mb) is a more powerful tool than the use of ROH of shorter lengths to analyze inbreeding depression [18].

The analysis including all the ROH showed a highly enriched annotation cluster including several precursors of the PAG family. These proteins are used as pregnancy-status indicators since their expression varies through pregnancy stages, and they are associated with placental mass, fetus number, and birth weight in cattle [64, 65]. Nowadays, the detection of PAG proteins is being used as an early pregnancy test in beef [66] and dairy cattle [67, 68], and also as an early marker of twin pregnancies [69]. Recently, the same protein family has been associated with milk yield, clinical diseases [70], pregnancy loss [71] and retained membranes [72]. In our study, PAG catabolism is the most significant pathway affected in inbreed individuals, suggesting that the reduced fertility observed in inbred bulls of this breed [73] could be partially mediated by the metabolism of those proteins.

In cattle, inbreeding has been also associated with changes in the immune system [74] and an increased occurrence of respiratory diseases [75]. Our results also identified a metabolic pathway related to B-defensin genes. Those proteins have been associated with embryos that showed a delayed developmental stage at day 7 after insemination [76] and with cows suffering *Staphylococci* mastitis [77]. Retarded embryos are often low quality and less viable embryos [78, 79], as well as mastitis has been recently associated with an impaired developmental competence in oocytes [80], even in sub-clinical presentations [81]. Both cases could also partially explain the mechanisms involved in the reduction of fertility observed in inbred individuals [73].

The analysis of the genomic positions affected by recently formed ROH showed three enriched clusters, but with a lower significance compared with the previous analysis. The most affected cluster was associated with microtubule structures, flagellum assembly, inositol polyphosphate kinase activity, chemotaxis and ATP-binding features. This cluster included the *DNAAF1*, *DNAH1*, *LRRC6* and *ZMYND10* genes. All those processes have been related to an impaired axonal assembly of the dynein arms that produced an abnormal movement of flagellum and cilia [82, 83]. Recently, mutations in *DNAH1* have been heavily associated with dysplasia of the sperm fibrous sheet [84], several flagellar defects and asthenozoospermia [85] in humans. These findings agree with a previous study which demonstrated that highly inbred Retinta bulls present increased hyperactivation-like motility and a reduced reproductive performance in field conditions [73]. We hypothesize that the accumulation of deleterious homozygote variants through excessive inbreeding events may have triggered this biological condition.

## Conclusion

In this study, we analyzed the use of F_ROH_ as IBD predictor in extremely inbred Retinta bulls. We showed that although it is correlated to the pedigree-based inbreeding coefficient (F_PED_), ROH seemed to distribute in an uneven fashion among individuals with similar F_ROH_ and F_PED_ coefficients. Furthermore, we found that individuals with a recent history of high inbreeding showed an increased number of long ROH (>8Mb). In this population, specific regions of the genome showed high accumulation of ROH, which was not associated to the recombination rate. ROH harbored genes related to pregnancy-associated proteins, cell motility, skeletal reorganization and immune system. These results are in line with a previous study where we showed that some of these bulls presented an increased hyperactivation-like motility pattern. Additionally, we demonstrated that animals with the same inbreeding coefficient may present different phenotypes, which may not always lead to a detriment in the production trait of interest.

## Supporting information

S1 Table ROH identification parameters at each length size per chromosome. **Footnote:** L: minimum number of SNPs; nH: number of heterozygous genotypes allowed per ROH; nM: number of missing genotypes allowed per ROH.
(DOCX)

S2 Table Descriptive statistics of ROH occurrence per chromosome and group of individuals. **Footnote:** Statistical differences were estimated between groups per chromosome using a T test.
(DOCX)

S3 Table Descriptive statistics of ROH length per chromosome and group of individuals. **Footnote:** Statistical differences were estimated between groups per chromosome using a T test.
(DOCX)

S4 Table Correlation between recombination rate and ROH abundance per chromosome. **Footnote:** Data was analyzed by chromosome in the whole population (WP) as well in HI and LI separately, using ROH abundance of any length or ROH>8Mb. Spearman correlation. Correlations were estimated per SNP within chromosome.
(DOCX)
