# Supplementary material for: Runs of homozygosity in a selected cattle population with extremely inbred bulls: Descriptive and functional analyses revealed highly variable patterns
Source: PLoS One. 2018 Jul 9;13(7):e0200069. doi: 10.1371/journal.pone.0200069 (PMC6037354; doi:10.1371/journal.pone.0200069)
Supplement: S1 Table — Footnote: L: minimum number of SNPs; nH: number of heterozygous genotypes allowed per ROH; nM: number of missing genotypes allowed per ROH. (DOCX) [file pone.0200069.s001.docx]

**S1 Table:** Parameters to identify ROH per chromosome with different minimum lengths.

 L: minimum number of SNPs; nH: number of heterozygous genotypes allowed per ROH; nM: number of missing genotypes allowed per ROH.
