# Supplementary material for: Runs of homozygosity in a selected cattle population with extremely inbred bulls: Descriptive and functional analyses revealed highly variable patterns
Source: PLoS One. 2018 Jul 9;13(7):e0200069. doi: 10.1371/journal.pone.0200069 (PMC6037354; doi:10.1371/journal.pone.0200069)
Supplement: S2 Table — Footnote: Statistical differences were estimated between groups per chromosome using a T test. (DOCX) [file pone.0200069.s002.docx]

**S2 Table**. Descriptive statistics of ROH occurrence per chromosome and group of individuals.

Statistical differences were estimated between groups per chromosome using a T test.
