# Supplementary material for: Runs of homozygosity in a selected cattle population with extremely inbred bulls: Descriptive and functional analyses revealed highly variable patterns
Source: PLoS One. 2018 Jul 9;13(7):e0200069. doi: 10.1371/journal.pone.0200069 (PMC6037354; doi:10.1371/journal.pone.0200069)
Supplement: S4 Table — Footnote: Data was analyzed by chromosome in the whole population (WP) as well in HI and LI separately, using ROH abundance of any length or ROH>8Mb. Spearman correlation. Correlations were estimated per SNP within chromosome. (DOCX) [file pone.0200069.s004.docx]

**S4 Table:** Correlation between recombination rate and ROH abundance per chromosome.

Data was analyzed by chromosome in the whole population (WP) as well in HI and LI separately, using ROH abundance of any length or ROH>8Mb. Spearman correlation. Correlations were estimated per SNP within chromosome.
